# Surgery or embolization for varicoceles in subfertile men

**DOI:** 10.1590/S1516-31802013000100014

**Published:** 2013-02-01

**Authors:** Anja C. J. Kroese, Natascha M. de Lange, John Collins, Johannes L. H. Evers

## Abstract

**BACKGROUND:**

A varicocele is a meshwork of distended blood vessels in the scrotum, usually left-sided, due to dilatation of the spermatic vein. Although the concept that a varicocele causes male subfertility has been around for more than 50 years now, the mechanisms by which a varicocele would affect fertility have not yet been satisfactorily explained. Neither is there sufficient evidence to explain the mechanisms by which varicocelectomy would restore fertility. Furthermore, it has been questioned whether a causal relation exists at all between the distension of the pampiniform plexus (a network of many small veins found in the human male spermatic cord) and impairment of fertility.

**OBJECTIVES:**

To evaluate the effect of varicocele treatment on live birth and pregnancy rate in subfertile couples where the male has a varicocele.

**METHODS:**

*Search*: We searched the Cochrane Menstrual Disorders and Subfertility Group Trials Register (12 September 2003 to January 2012), the Cochrane Central Register of Controlled Trials (CENTRAL) in The Cochrane Library Issue 1, 2012), Medline (January 1966 to January 2012), Embase (January 1985 to January 2012), PsycINFO (to Week 1 2012) and reference lists of articles. In addition, we handsearched specialist journals in the field from their first issue until 2012. We also checked cross-references, references from review articles and contacted researchers in the field.

*Selection criteria:* Randomized controlled trials (RCTs) were included if they were relevant to the clinical question posed. If they reported pregnancy rates or live birth rates as an outcome measure, and if they reported data in treated (surgical ligation or radiological embolization of the internal spermatic vein) compared to untreated or placebo groups. Two authors independently screened potentially relevant trials. Any differences of opinion were resolved by consensus (none occurred for this review).

*Data collection and analysis:* Ten studies met the inclusion criteria for the review. For one study we had only data from a published abstract. All ten studies only included men from couples with subfertility problems; one excluded men with sperm counts less than 5 million per mL and one excluded men with sperm counts less than 2 million per mL, with or without progressive motility of less than 10%. Two trials involving clinical varicoceles included some men with normal semen analysis. Three studies specifically addressed only men with subclinical varicoceles. Studies were excluded from meta-analysis if they made comparisons other than those specified above.

**MAIN RESULTS:**

The meta-analysis included 894 men. No studies reported live birth. The combined fixed-effect odds ratio (OR) of the 10 studies for the outcome of pregnancy was 1.47 (95% confidence interval (CI) 1.05 to 2.05, very low quality evidence), favouring the intervention. The number needed to treat for an additional beneficial outcome was 17, suggesting benefit of varicocele treatment over expectant management for pregnancy rate in subfertile couples in whom varicocele in the man was the only abnormal finding. Omission of the studies including men with normal semen analysis and subclinical varicocele, some of which had semen analysis improvement as the primary outcome rather than live birth or pregnancy rate, was the subject of a planned subgroup analysis. The outcome of the subgroup analysis (five studies) also favoured treatment, with a combined OR 2.39 (95% CI 1.56 to 3.66). The number needed to treat for an additional beneficial outcome was 7. The evidence was suggestive rather than conclusive, as the main analysis was subject to fairly high statistical heterogeneity (I2 = 67%) and findings were no longer significant when a random-effects model was used or when analysis was restricted to higher quality studies.

**AUTHORS’ CONCLUSIONS:**

There is evidence suggesting that treatment of a varicocele in men from couples with otherwise unexplained subfertility may improve a couple’s chance of pregnancy. However, findings are inconclusive as the quality of the available evidence is very low and more research is needed with live birth or pregnancy rate as the primary outcome.

**This is the abstract of a Cochrane Review published in the Cochrane Database of Systematic Reviews (CDSR) 2012, issue 10, DOI:** 10.1002/14651858.CD000479.pub1 (http://onlinelibrary.wiley.com/doi/10.1002/14651858.CD000479.pub5/abstract). For full citation and authors details see reference 1.

**The full text is freely available for Latin America and the Caribbean from:** http://cochrane.bvsalud.org/cochrane/show.php?db=reviews&mfn=357&id=CD000479&lang=pt&dblang=&lib=COC&print=yes
